# Raman Spectroscopy Monitoring of Duck Egg Brining Process

**DOI:** 10.3390/foods13233775

**Published:** 2024-11-25

**Authors:** Huaizhou Jin, Yanxia Zou, Shangzhong Jin, Qiang Lin

**Affiliations:** 1 Key Laboratory of Quantum Precision Measurement, College of Physics, Zhejiang University of Technology, Hangzhou 310014, China; jinhuaizhou@zjut.edu.cn (H.J.); qlin@zjut.edu.cn (Q.L.); 2 College of Optical and Electronic Technology, China Jiliang University, Hangzhou 310018, China; zouyanxia92@163.com

**Keywords:** salted duck eggs, Raman spectroscopy, salting, food processes

## Abstract

Salted duck eggs are a popular food in China and a key ingredient in pastries such as mooncakes, valued for their unique flavors. In this study, we examined the influence of brining processes on duck eggs, focusing on salt concentration and the effect of added wine. Four experimental groups were established: 18% salt, 25% salt, and 18% or 25% salt with added wine. The results from egg yolks suggest that increasing the salt concentration or adding 10% wine (53% alcohol) accelerates the brining process, while the Raman spectra of egg whites remain remarkably stable throughout brining. Our findings suggest that the traditional 30-day brining period can be reduced to 20–25 days with a higher salt concentration or the addition of wine, after which the egg yolk structure becomes largely stable.

## 1. Introduction

Eggs are widely used in breakfasts, home meal preparation, cooking, and as an ingredient in many foods due to their richness in proteins, lipids, and essential vitamins and minerals [1]. They also have a wide range of functional food properties including solubility, water retention capacity, emulsification, fat binding, foaming, and gelling [2]. Hen and duck eggs are the most consumed egg products because they provide complete proteins containing all essential amino acids for humans, along with several vital vitamins and minerals. Moreover, eggs are one of the most affordable single food sources of complete protein [3,4].

To maintain the quality and extend the shelf life of eggs, processing and preservation methods are necessary. One common preservation technique is salting, which primarily uses sodium chloride as a preservative [5]. Sodium chloride is key in reducing the growth of internal microorganisms and prolonging the shelf life of eggs. During the salting process, sodium chloride gradually diffuses into the egg white and yolk through the pores and membranes of the shell [6]. Because of the unique properties and flavor of salted eggs, salted egg yolk is widely used as ingredient in pastries and bakery products, such as mooncakes [7].

Salted eggs are generally made from duck eggs due to their desirable properties compared to hen eggs [8]. Duck eggs have several advantages that make them more suitable for salted egg production. Firstly, they have larger yolks and more albumen than hen eggs, which facilitates easier production and results in a richer yolk portion that enhances the flavor of the salted egg [9]. Secondly, duck eggs secrete more oil, further contributing to the taste. The shell of a duck egg is also harder and more resistant to damage during storage and transportation, making it a more durable choice. Furthermore, a duck egg’s shell has a higher density of holes per square centimeter on average compared to a hen egg’s shell, allowing for better salt diffusion into the egg [10]. There are two common methods used in the egg-salting process: brining and dry-curing [6]. The former refers to the method where the eggs are soaked in salt solutions, which may or may not contain alcohol, whereas the latter refers to the method where the eggs are packed in damp and salted charcoal.

In this study, we investigate the former method, brining. This method was chosen because it offers better hygiene control and more precise monitoring of preservation conditions than dry-curing. Brining is also easier to standardize, which means our findings can be more readily applied to improve commercial production processes.

A duck egg consists of a shell with a horny (keratinous) layer, egg white, and yolk. Duck egg white makes up 60% of the whole egg; it consists of approximately 88% water and about 11% protein, with the remaining 1% consisting of minerals, trace amounts of fats, and glucose [11,12]. The proteins in egg whites primarily include ovalbumin (~54%), ovotransferrin (~12%), ovomucoid (~11%), lysozyme (~3.5%), ovomucin (~3.5%), and globulins (~8%) [13]. The yolk, an emulsified liquid, is the central part of the duck egg. The yolk is primarily composed of 50% water, 15 to 17% proteins, and 31% to 33% lipids; lipids include triglycerides (~65%), phospholipids (~31%), cholesterol (~4%), and trace amounts of minor lipids such as ceruloplasmin or glycolipids [13,14]. Egg yolks also contain pigments (carotenoids and riboflavin), which are responsible for regulating the color of the yolk. Finally, egg yolks contain minerals, with phosphorus having the highest content [11].

Raman spectroscopy is a powerful tool in food science due to its non-destructive nature and ability to provide detailed molecular structural information [15]. In food science, Raman spectroscopy was used successfully to monitor various types of food processes, including protein denaturation and lipid oxidation [16,17]. Recent advances in Raman spectroscopic methods also allow it to offer widespread food safety assessment in a non-destructive, sensitive, and rapid manner [18]. Surface-enhanced Raman spectroscopy is also being used to detect toxins, bacteria, or contaminants in food [19,20,21].

There are other analytical and spectroscopic methods used to study food processes such as salting. For example, ultrasonic spectroscopy is often used to monitor the dry-salting of meat [22,23], and Fourier-transform infrared (FTIR) spectroscopy was used to monitor the salting process of salmon [24,25]. However, Raman spectroscopy offers several advantages. It is rapid, requires minimal sample preparation, and most importantly, has very weak water interference, whereas both ultrasonic and FTIR spectroscopy are affected by water content. This makes Raman spectroscopy especially suitable for the study of duck eggs, which contain about 70% water.

In this study, we present a comprehensive Raman spectroscopic analysis of the duck egg brining process, monitoring structural changes in both egg whites and yolks over time. Four different brining solutions were prepared to systematically investigate the effects of salt concentration and wine addition on the molecular-level changes during brining. Traditional salted duck egg production relies on empirical knowledge and extended brining periods; however, Raman spectroscopy, supported by advanced spectral preprocessing methods, offers a new analytical framework for understanding the actual timeline of structural modifications. Through quantitative analysis of characteristic peak intensities, this study aims to establish a scientific basis for optimizing brining duration and conditions in salted duck egg production.

## 2. Materials and Methods

### 2.1. Experimental Design

This study investigated the effects of salt concentration and Chinese wine (baijiu) addition on duck egg brining using Raman spectroscopy at room temperature (25 °C). Four groups were used:

- L1: 18% salt solution;
- L2: 18% salt solution with 10% baijiu;
- L3: 25% salt solution;
- L4: 25% salt solution with 10% baijiu.

The salt concentrations were carefully selected based on both practical and technical considerations. The 18% concentration was chosen as it represents the midpoint of traditional brining concentrations (typically ranging from 16.7% to 20%), while 25% was selected as it approaches but remains slightly below the salt saturation point at room temperature, ensuring solution stability throughout the brining period. The addition of 10% baijiu was based on traditional Chinese culinary practices, where the alcohol is believed to enhance flavor development through improved penetration of salt into the eggs.

### 2.2. Materials

The duck eggs, salt, and wine used in this study were purchased from a local supermarket. Each egg weighed between 65 g and 85 g; eggs of different sizes were distributed evenly between the four brining conditions. The salt used was food-grade refined iodized salt. Chinese baijiu (a type of Chinese wine commonly used for drinking or for brining and fermentation purposes) was added to two groups of brining solutions; the baijiu used in this study contains 53% alcohol. Sealed pickle jars were used as containers to pickle the duck eggs, and the water used for brining was tap water from China Jiliang University’s laboratory floor.

### 2.3. Sample Preparation

In this study, a total of four brining solutions were prepared: 18% salt solution (L1), 18% salt solution with 10% added baijiu (L2), 25% salt solution (L3), and 25% salt solution with 10% added baijiu (L4). First, the two salt solutions (18% and 25%) were prepared separately. These salt solutions were then boiled and filtered to remove microorganisms such as bacteria, viruses, and parasites from the water. Boiling also ensured that the food-grade salt solids were thoroughly dissolved in the water, improving the homogeneity of the salt solutions. After boiling, the salt solutions were placed in an air-conditioned room to cool. Once cooled, baijiu was added to the corresponding salt solutions to create the L2 and L4 brining solutions, respectively.

Fresh duck eggs were cleaned and treated before brining. First, the surface dirt on the fresh duck eggs was manually cleaned and removed, and water stains on the surface were wiped away. Next, the cleaned duck eggs were dried under room temperature for one hour. After drying, the duck eggs were equally divided into four groups and placed into the L1, L2, L3, and L4 brining solutions to begin the brining process at room temperature. The weight ratio of duck eggs to brining solution was maintained at 1:1. The room temperature for both drying and brining was maintained at 25 °C.

### 2.4. Raman Experiments

Data Collection: Raman spectra were collected at 5-day intervals over a 40-day period. On each sampling day, four eggs from each treatment group (L1, L2, L3, and L4) were analyzed. For each egg, Raman spectra were collected at 5 points in the egg white and 5 points in the egg yolk, yielding 20 spectra per component (white/yolk) per treatment group per day. As a baseline, four fresh eggs were analyzed following the same protocol prior to brining (day 0).

Before testing, the duck eggs were first removed from the brining solutions, and the water stains on the surface were wiped clean. The eggshells were then cracked, and the egg white and yolk contents were carefully separated and placed into labeled centrifuge tubes, ensuring that the whites and yolks from different eggs were kept separate.

To prepare the samples for Raman spectroscopy, the whites or yolks in each centrifuge tube were thoroughly stirred to ensure homogeneity and then placed on a clean glass slide. All measurements were performed using a Horiba LabRAM HR Evolution Raman spectrometer (Horiba Jobin Yvon, Kyoto, Japan) with a 50× long-focal-length objective (NA = 0.75). Egg yolks were measured using a 633 nm laser at 100% power (spot diameter ~1.03 μm), while egg whites were measured using a 532 nm laser at 10% power (spot diameter ~0.86 μm). For both samples, spectra were acquired with a 600 grooves/mm grating, 30 s exposure time, and two accumulations to improve the signal-to-noise ratio. The spectral resolution was approximately 3–4 cm^−1^ and 2–3 cm^−1^ for yolks and whites, respectively.

The Raman system was wavelength-calibrated using a silicon wafer (520.7 cm^−1^). For each sample, the Raman spectra were collected for 30 s and accumulated twice to improve the signal-to-noise ratio.

### 2.5. Raman Spectral Pre-Processing for Egg Yolk Spectra

Spectral pre-processing, most notably baseline correction and smoothing, is necessary when obtaining the Raman spectra of eggs. Egg whites and yolks are complex biological matrices containing various proteins, lipids, and other biomolecules, which can contribute to intrinsic fluorescence and background noise in the Raman spectra. These interfering signals can obscure the true Raman peaks and make it difficult to identify and quantify the chemical components of interest.

Smoothing is used to reduce the noise and improve the signal-to-noise ratio of the spectral signal. Smoothing methods commonly used for Raman spectra include moving window averaging, Savitzky–Golay filtering (SG filtering), Whittaker smoother, and wavelet transform [26]. The primary function of baseline correction is to reduce or remove fluorescence backgrounds. Typical baseline correction methods include polynomial fitting, wavelet transform, spline curve fitting, iterative smoothing, and penalized least squares [27,28].

In this study, the algorithms of SG filtering (window n = 7; the polynomial order is second-order) and adaptive iterative reweighted penalized least squares (air-PLS) with higher accuracy [29] were implemented using MATLAB R2024a to successively complete the smoothing and baseline calibration processing of the raw Raman data for the duck eggs. To ensure that fluctuations in Raman intensity do not impact the results, the spectra were also normalized. The egg yolk spectra were normalized using the 2852 cm^−1^ CH_2_ peak as a reference, whereas the egg white spectra were normalized using the 1004 cm^−1^ symmetric ring breathing/phenylalanine peak as reference.

## 3. Results

### 3.1. Peak Assignment of Duck Egg Spectra

During the brining process, the moisture, protein, lipid, and other components within duck eggs undergo temporal changes, contributing to the distinctive quality and flavor profile of salted duck eggs. Numerous studies have employed various analytical methods to monitor these changes, focusing on parameters such as lipid content, moisture levels, and yolk hardening [30,31]. In this study, changes to Raman spectral peaks during brining were analyzed. Here, Table 1 presents the peak assignments for the most prominent Raman bands observed in the spectra of duck egg whites and yolks.

During the brining process, while the peak positions mostly remain unchanged, the intensity ratio between different peaks changes, showing alterations in the relative concentrations of various molecular components within the duck eggs. These spectral changes reflect the complex biochemical transformations occurring during brining, such as protein denaturation, lipid content alteration, and salt penetration.

### 3.2. Spectral Changes During the Brining Process for Egg Yolks

The flavor, quality, and stability of salted duck eggs are significantly influenced by variations in curing environments and durations [34,35,36]. We first examine the spectral changes to egg yolks during the brining process. As discussed in the previous section, the yolk spectra were normalized using the 2852 cm^−1^ CH_2_ peak as the reference.

**Figure 1:**
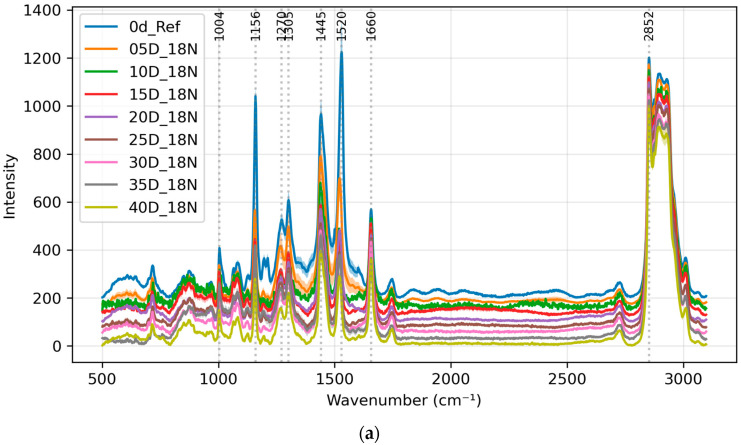

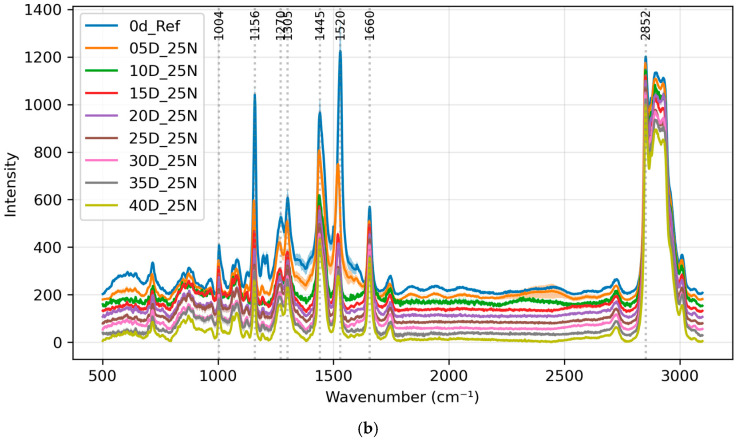
Averaged spectra of salted egg yolks from day 0 to day 40 for groups (**a**) L1 and (**b**) L3. The labeled peaks from left to right are 1004, 1156, 1270, 1305, 1445, 1520, 1660 and 2852cm^−1^, respectively. Offset added for clarity. Top to bottom is from day 0 to day 40 for both figures.

The L1 group was first used as an example. During the brining process, the intensity of other lipid peaks, such as 1660 cm^−1^ and 2913~2938 cm^−1^, did not have significant changes with respect to the 2852 cm^−1^ CH_2_ peak. This suggests that the overall lipid content and structure are relatively unchanged during brining. This result is the same for the L3 group. Figure 1 shows the spectral progression from 0 days (before brining) to 40 days for groups L1 and L3, where each spectrum represents the mean of 20 measurements (5 spots per egg × 4 eggs) taken under identical conditions (same day and treatment group). The labels of the figures in this manuscript are explained in Appendix A.

Notable changes were observed in the Raman spectra at 1445 cm^−1^. The 1445 cm^−1^ peak is relatively wide and contains two peaks: the cholesterol and CH_2_ scissoring peak at 1440 cm^−1^ and the collagen and phospholipids peak at 1455 cm^−1^. This peak showed a significant decrease in intensity relative to the reference 2852 cm^−1^ CH_2_ peak during the salting process for both the L1 (18% salt) and L3 (25% salt) groups.

Possible reasons for this change may include alterations in cholesterol content or conformation, structural modifications of phospholipids and collagen, or changes in their interactions with the surrounding environment due to salt-induced dehydration. The relative increase in lipid-related peaks at 2852 cm^−1^, 1660 cm^−1^, and 2913~2938 cm^−1^ could also be attributed to a concentration effect as water is drawn out of the yolk, or to conformational changes in lipids leading to enhanced Raman signals.

**Figure 2:**
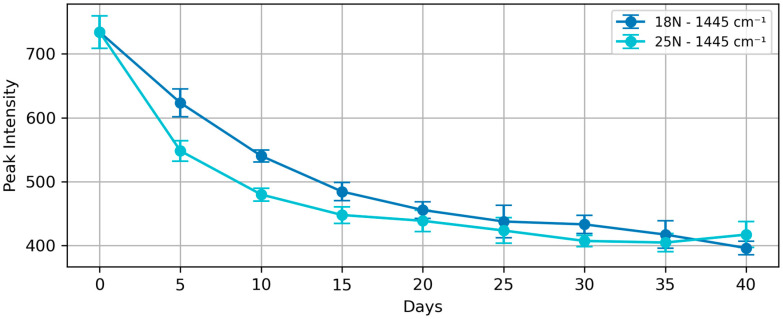
Trend of 1445 cm^−1^ peak (relative to 2852 cm^−1^ peak) for groups L1 (labeled 18N) and L3 (labeled 25N), with error bars representing the standard deviation (SD) calculated from 20 measurements per data point (n = 20). The L3 group has a notably sharper decline in the 1445 cm^−1^ intensity in the first 15 days. All error bars in subsequent figures represent SD unless otherwise specified.

Figure 2 shows the trend of the 1445 cm^−1^ peak intensity when the 2852 cm^−1^ peak is normalized to 1000 a.u. intensity for groups L1 and L3. The L3 group (25% salt) shows a more rapid initial decrease compared to the L1 group (18% salt), especially in the first 15 days. This faster rate of change in the L3 group indicates that higher salt concentrations accelerate the structural modifications in egg yolk lipids during the early stages of the process.

The difference between the L1 and L3 groups is most pronounced during days 5 to 15, where the separation between curves is statistically significant as demonstrated by non-overlapping error bars (*p* < 0.05). After day 20, structural modifications slow down considerably as the system approaches equilibrium, and error bars start to overlap. Both groups converge to similar values around 400 to 450 a.u.

Both the L1 and L3 groups reach a plateau around 20–25 days and converge to similar end-point values for the 1445 cm^−1^ peak intensity (around 400 to 450 a.u.). This indicates that while salt concentration affects the rate of the salting process, especially in the early stages, it does not significantly alter the final equilibrium state of lipid structures in the egg yolk.

**Figure 3:**
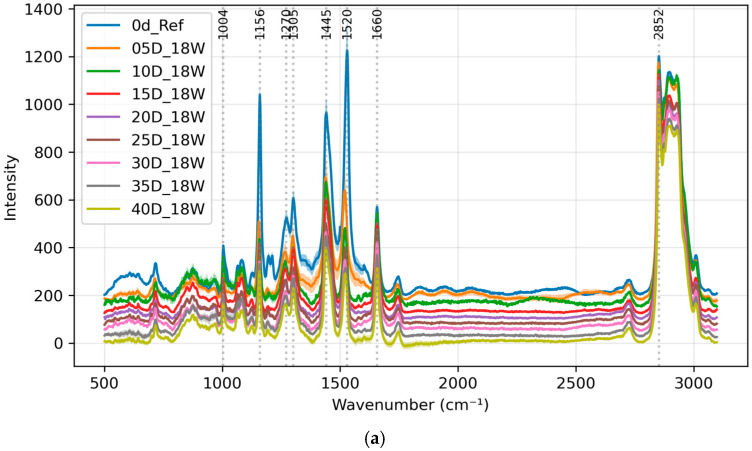

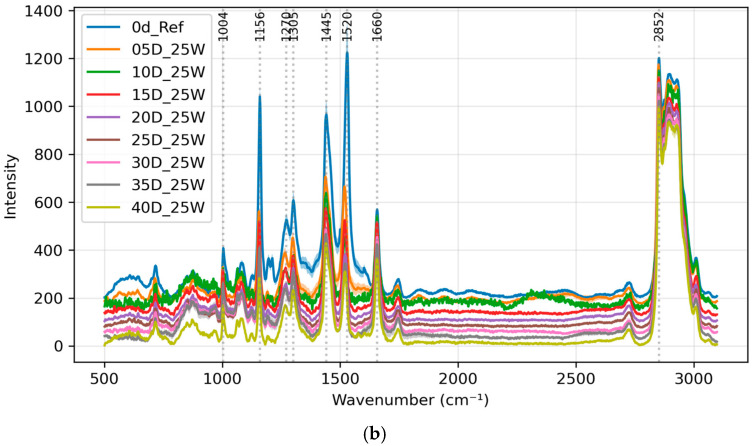
Averaged spectra of salted egg yolks from day 0 to day 40 for groups (**a**) L2 and (**b**) L4. The labeled peaks from left to right are 1004, 1156, 1270, 1305, 1445, 1520, 1660 and 2852cm^-1^, respectively. Offset is added for clarity. Top to bottom is from day 0 to day 40 for both figures.

**Figure 4:**
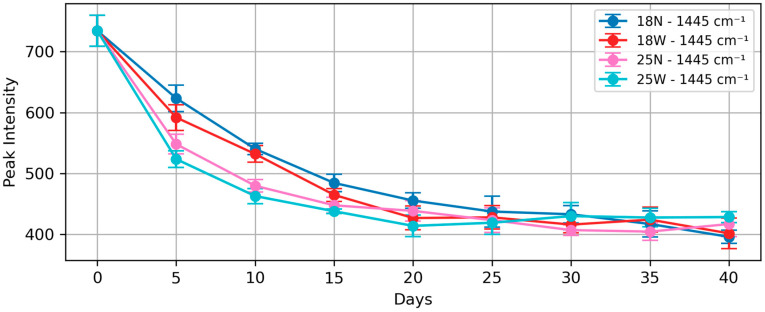
Trend of 1445 cm^−1^ peak (relative to 2852 cm^−1^ peak) for all four groups with error bars: 18N and 25N are groups L1 and L3, meaning 18% and 25% salt concentration without added wine; 18W and 25W means groups L2 and L4, meaning 18% and 25% salt concentration with added wine.

The effect of added wine is examined with groups L2 and L4. The averaged spectra of the L2 and L4 groups from day 0 to day 40 are shown in Figure 3. The trend of the 1445 cm^−1^ peak for all four groups is shown in Figure 4. Groups L2 and L4 are the groups with wine added during the brining process. The first derivative spectra of all four groups are also shown in Figure S1.

A rapid decrease in peak intensity is observed during the first 15 days for all groups. During the first 15 days, the presence of alcohol appears to accelerate the salting process; however, the effect of higher salt concentration is more significant than that of added wine. The trend of the L2 group (18W in Figure 4) sits mostly below the trend of the L1 group (18N in Figure 4), and the trend of the L4 group also sits mostly below that of the L3 group. For all four groups, the changes to the 1445 cm^−1^ peak start to slow down from day 15 onwards.

The enhanced rate of change in the presence of wine could be attributed to the alcohol content facilitating salt penetration into the egg yolk, or to organic compounds in the wine interacting with yolk lipids to promote structural changes. Despite the faster kinetics, the wine-added groups appeared to approach similar end-point values for the 1445 cm^−1^ peak.

We then investigated the effect of brining on anti-oxidants in egg yolks. The trends of carotenoid peaks at 1156 and 1520 cm^−1^ are shown in Figure S2. The results show that in the initial stages of brining the intensity of the carotenoid peaks decreases, with a very sharp decrease from day 0 to 5, and then a smaller decrease from day 5 to 10. Both trends are then followed by fluctuations around relatively stable mean values. This may suggest that major changes in carotenoid structure occur within the first 10 days, after which the protective effect of brining appears to stabilize the system. The subsequent fluctuations likely represent experimental variations.

Last but not least, there also does not appear to be any extra ethanol-specific peak (such as at 883 cm^−1^) in either group L2 or L4 in Figure 3 compared to groups L1 and L3 in Figure 1. This means no ethanol residues were detected using Raman spectroscopy.

### 3.3. Spectral Changes During the Brining Process for Egg Whites

In the previous section, egg yolks showed significant spectral changes during brining. We next examined whether similar modifications occurred in egg whites.

**Figure 5:**
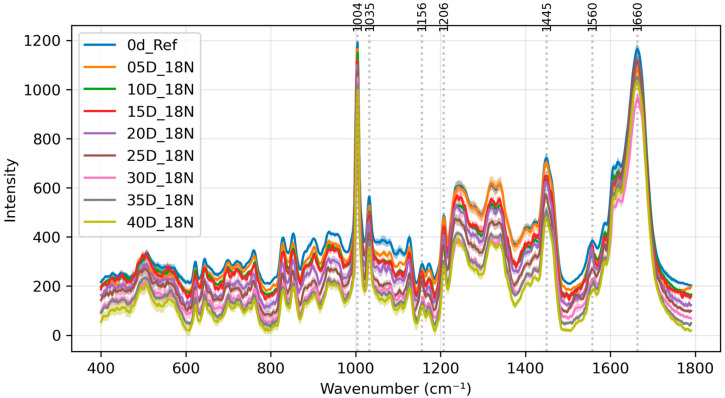
Averaged spectra of salted egg whites from day 0 to day 40 for group L1. Offset is added for clarity. Top to bottom is from day 0 to day 40.

Figure 5 shows the averaged spectra of the egg whites of group L1 from day 0 to day 40 with shaded error regions. The averaged spectra of every testing day, when normalized with respect to the 1004 cm^−1^ peak, remain consistent from day 0 to day 40 with minimal changes. The peak positions stay consistent over the entire brining process, and the relative intensity of each peak remains stable. The averaged spectra of egg whites of groups L2, L3, and L4 over the 40-day brining process are very similar to Figure 5 and are shown in Figure S3. The first derivative spectra of the egg whites of all groups from day 0 to day 40 are shown in Figure S4.

Egg whites primarily consist of water (88%) and proteins (11%), with ovalbumin being the most abundant protein. In order to monitor potential structural changes during brining and verify the spectral consistency over the brining process, we focused on three characteristic protein peaks that are commonly used to assess protein conformational changes. These three protein peaks are Amide I at 1660 cm^−1^ (C=O stretching), CH_2_/CH_3_ deformation at 1445 cm^−1^ (protein side chains), and Amide III at 1305 cm^−1^ (C-N stretching and N-H bending). Unlike egg yolks, where the 1445 cm^−1^ peak includes contributions from both proteins and lipids, in egg whites, this peak primarily reflects protein side chain vibrations due to the negligible lipid content.

**Figure 6:**
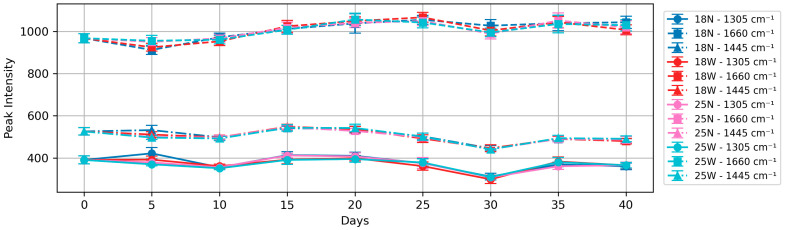
Trend of 1305, 1445, and 1660 cm^−1^ peaks (relative to 1004 cm^−1^ peak) for all four groups with error bars; 18N, 18W, 25N, and 25W are groups L1 to L4, respectively.

The trends of the three protein peaks are shown in Figure 6. All three characteristic protein peaks remain consistent over the 40-day brining process, regardless of salt concentration or the addition of wine. This stability contrasts markedly with egg yolks, which undergo obvious structural changes throughout the brining process, suggesting that the protein structures in egg whites experience minimal modification during brining.

The stability of the egg white spectra in all four groups suggests that the brining process has relatively little effect on the molecular structures of proteins in egg whites. Brining causes dehydration and increased firmness in egg whites, and egg whites taste significantly saltier after brining. However, these physical and sensory changes appear to occur without substantial alterations to the protein structure.

## 4. Discussion

By investigating the 1445 cm^−1^ peak of egg yolks, which contains contributions from cholesterol, CH_2_ scissoring, collagen, and phospholipids, we found that the brining process causes this peak to decrease significantly compared to the 2852 cm^−1^ peak. This decrease suggests structural reorganization of lipids in the egg yolks, with both higher salt concentration and wine addition accelerating these changes. All four groups eventually converge to similar end-point values (400–450 a.u.) at around 20–25 days, indicating a common equilibrium state regardless of treatment conditions.

In contrast, the Raman spectra of egg whites show remarkable stability throughout the brining process. The Raman spectra of egg whites, including the three characteristic protein peaks (Amide I at 1660 cm^−1^, CH_2_/CH_3_ deformation at 1445 cm^−1^, and Amide III at 1305 cm^−1^), maintain consistent intensities across all treatment conditions. This suggests that the physical changes in egg whites during brining occur without significant alterations to protein structure. This stability persists regardless of salt concentration or wine addition.

In the spectra of both yolks and whites, adding wine does not appear to leave alcohol residues that can be detected with Raman spectroscopy. The carotenoid peaks at 1156 and 1520 cm^−1^ also suggest that brining may have a protective effect in preserving carotenoids after the first 5–10 days.

The findings from this study may have important implications for commercial production. While traditional brining practices typically extend to 30 days, our spectral analysis suggests this duration could be reduced by up to 10 days when using a higher salt concentration or adding wine. However, further studies are required to ensure that the shortened brining process still produces salted duck eggs with optimal structural, taste, and texture properties.

Recent studies have also employed Raman spectroscopy to study egg properties, mostly with different focuses such as examining egg freshness, or the detection of rotten eggs or antibiotics. For example, Davari et al. used Raman spectroscopy to assess egg freshness by measuring both egg shells and yolks [37]; Tan et al. and Zhang et al. used surface-enhanced Raman spectroscopy (SERS) to detect hydrogen sulfide and antibiotic residues in eggs, respectively [38,39].

In contrast to these mostly assessment or detection focused studies, our work demonstrates Raman spectroscopy’s capability for continuous monitoring of the brining process through the 1445 cm^−1^ to 2852 cm^−1^ peak ratio in yolks. Because of the thick shell of duck eggs, non-invasive monitoring of the Raman spectra of duck eggs is challenging. Still, the spectral changes observed in this study are consistent. This means methods to monitor the brining, salting, or general curing of egg-based products can be established if the relationship between Raman intensity and the curing process can be revealed.

## 5. Conclusions

This study demonstrates that Raman spectroscopy can be used to effectively monitor molecular structural changes during the duck egg brining process. We observed that egg yolks undergo notable structural alterations as a result of brining, whereas the proteins in egg whites remain largely unchanged. Higher salt concentration (25% vs. 18%) and wine addition accelerate these structural changes, particularly in the first 10–15 days, as evidenced by changes in the 1445 cm^−1^ peak relative to the 2852 cm^−1^ peak. The structural modifications in egg yolks largely stabilize after 20–25 days of brining, suggesting that a higher salt concentration and/or added wine could potentially reduce the traditional 30-day brining period to 15–20 days. Additionally, the behavior of carotenoid peaks indicates an initial rapid change followed by stabilization, suggesting a potential protective effect of brining against further oxidation. These findings provide new insights into the molecular processes during egg brining and may have practical implications for optimizing commercial production processes.

## Figures and Tables

**Table 1:** Peak attribution of duck egg spectra [32,33].

Wavenumber	Assignment
718 cm^−1^	CN^−^(CH_3_)_3_ symmetric stretch (lipids)
875 cm^−1^	Characteristic for phospholipids
880 cm^−1^	Tryptophan, δ(ring)
1004 cm^−1^	ν_s_(C-C), symmetric ring breathing, phenylalanine (protein)
1084 cm^−1^	ν_s_(C-C) Phospholipid backbone
1156 cm^−1^	Carotenoids
1206 cm^−1^	Tyrosine (collagen)
1270 cm^−1^	=C-H in-plane deformation (phospholipids)
1305 cm^−1^	CH_2_ twisting (fatty acids)/proteins and phospholipids
1335 cm^−1^	CH_3_CH_2_ wagging mode of collagen
1440 cm^−1^	δ(CH_2_) scissoring (fatty acids, cholesterol)
1455 cm^−1^	δ(CH_2_) bending (collagen and phospholipids)
1520 cm^−1^	-C=C- (carotene)
1657 cm^−1^	ν(C=C) cis (phospholipids)
1660 cm^−1^	Amide I (C=O stretch)/C=C (lipids)
2852 cm^−1^	ν_s_(CH_2_) symmetric stretch (fatty acids)
2885 cm^−1^	ν_s_(CH_3_) symmetric stretch (fatty acids)
2913 cm^−1^	ν_as_(CH_2_) asymmetric stretch (fatty acids)
2938 cm^−1^	ν_as_(CH_3_) asymmetric stretch (fatty acids)

## Data Availability

The original contributions presented in the study are included in the article/Supplementary Material, further inquiries can be directed to the corresponding author.

## Supplementary Materials

The following supporting information can be downloaded at https://www.mdpi.com/article/10.3390/foods13233775/s1: Figure S1: First derivative spectra of egg yolks (averaged; with error shades) of all four groups. (**a**–**d**) represent groups L1 to L4, respectively.; Figure S2: Trends of carotenoid peaks at (**a**) 1156cm^−1^ and (**b**) 1520cm^−1^ in the brining duck eggs from day 0 to day 40.; Figure S3: Averaged spectra of salted egg whites from day 0 to day 40 for groups (**a**) L2, (**b**) L3 and (**c**) L4. Offset is added for clarity. Top to bottom is from day 0 to day 40 for all samples.; Figure S4: First derivative spectra of egg whites (averaged; with error shades) of all four groups. (**a**–**d**) represent groups L1 to L4, respectively.

## Appendix A

Here, we explain the labels in the figures in this manuscript as well as in the Supplementary Materials. In the spectral figures, offset is added to the spectra for clarity. The labels of the spectra are the filenames we used when acquiring the data. The prefixes of the labels (0D to 40D) are the days of brining (0D_Ref means before brining), and the suffixes include the salt concentration (18 or 25 means 18% or 25% salt concentration), with the final letter “W” meaning baijiu was added to the brining solution, and “N” meaning baijiu was not added. For example, 5D_18W means day 5, 18% salt concentration, and that baijiu was added to the brining solution. Additionally, data from day 0 to day 40 are typically arranged from top to bottom for all spectra figures.
