# Supplementary material for: Raman Spectroscopy Monitoring of Duck Egg Brining Process
Source: Foods. 2024 Nov 25;13(23):3775. doi: 10.3390/foods13233775 (PMC11640425; doi:10.3390/foods13233775)
Supplement: Supplementary file 1 [file foods-13-03775-s001.zip › foods-3274643-supplementary.pdf]

Support Information

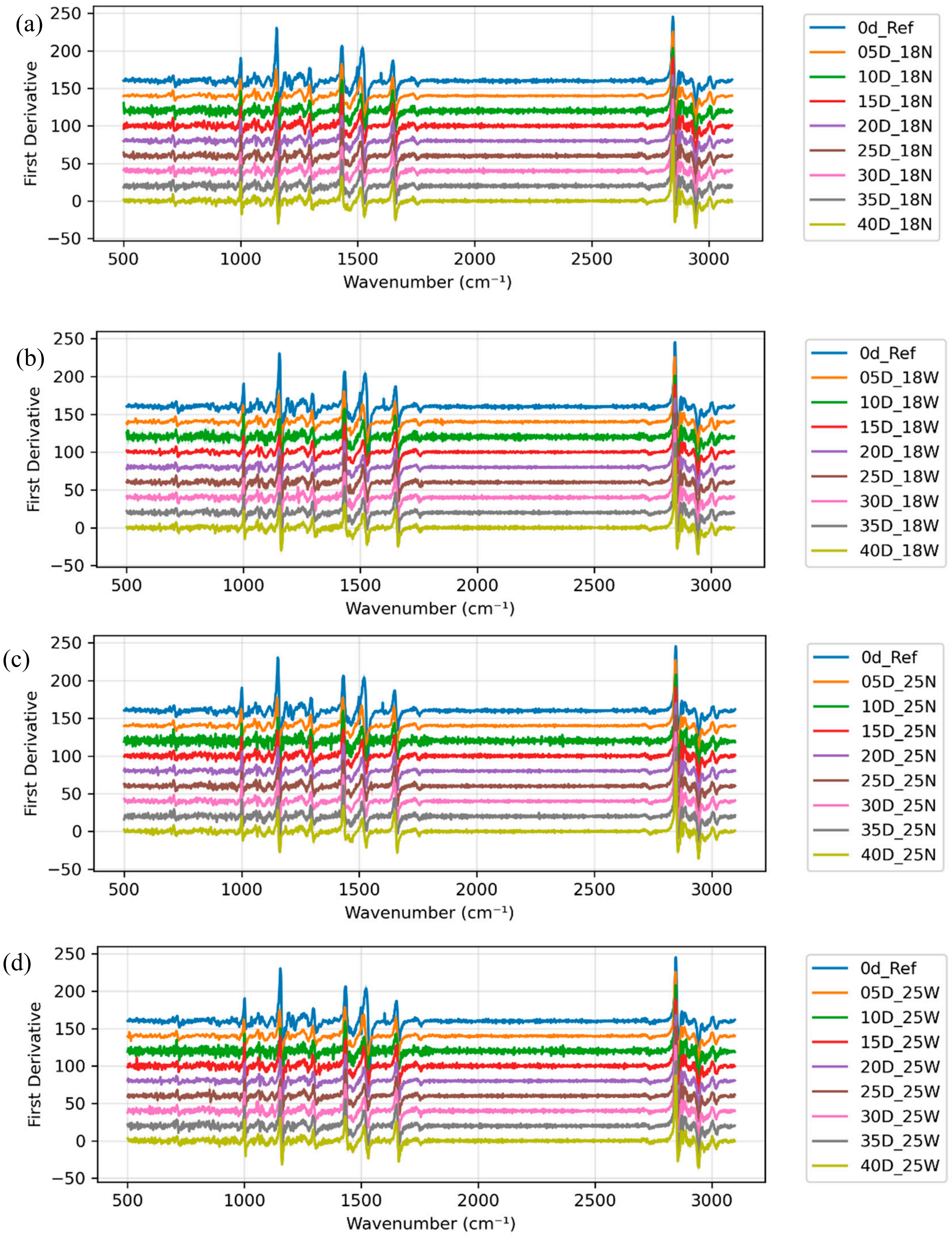

Figure S1 First derivative spectra of egg yolks (averaged; with error shades) of all four groups. (**a**–**d**) represent groups L1 to L4, respectively

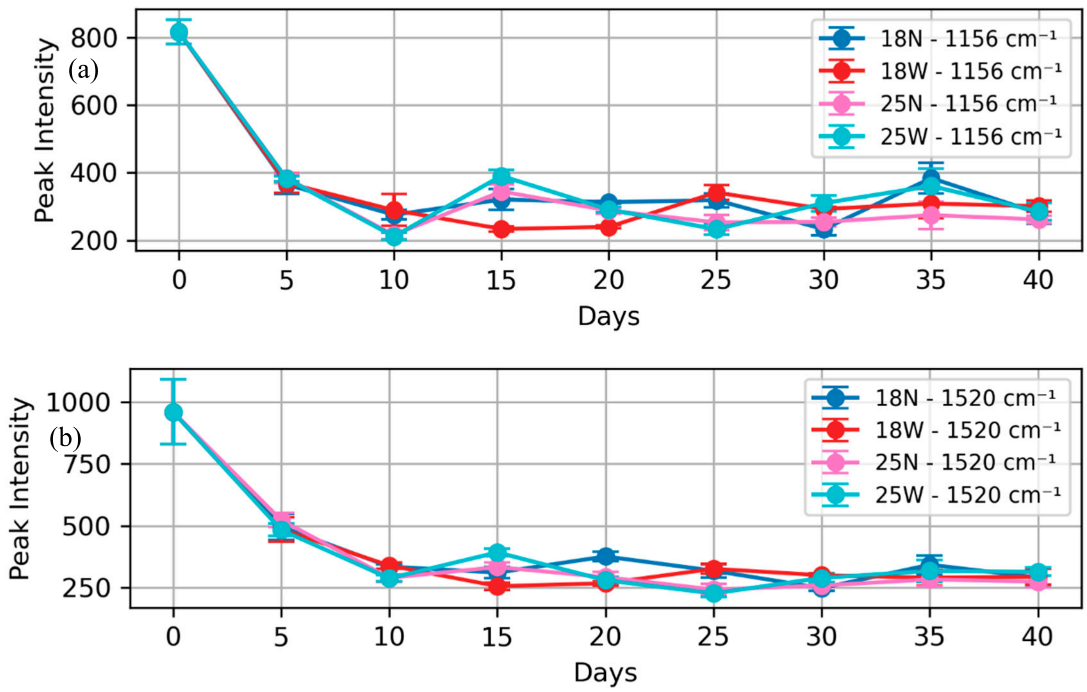

Figure S2 Trends of carotenoid peaks at (**a**) 1156cm^{-1} and (**b**) 1520cm^{-1} in the brining duck eggs from day 0 to day 40

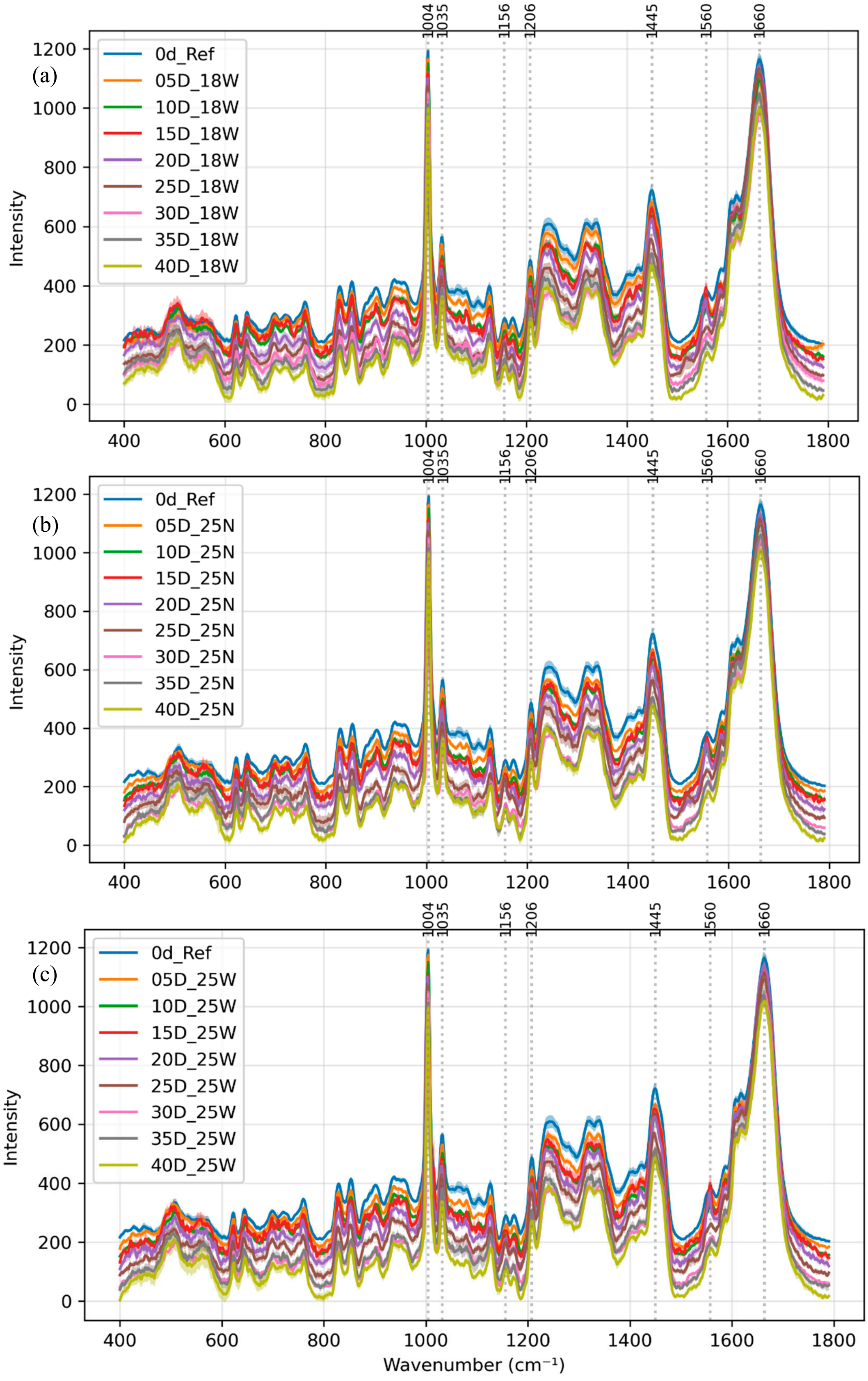

Figure S3 Averaged spectra of salted egg whites from day 0 to day 40 for groups (**a**) L2, (**b**) L3 and (**c**) L4. Offset is added for clarity. Top to bottom is from day 0 to day 40 for all samples.

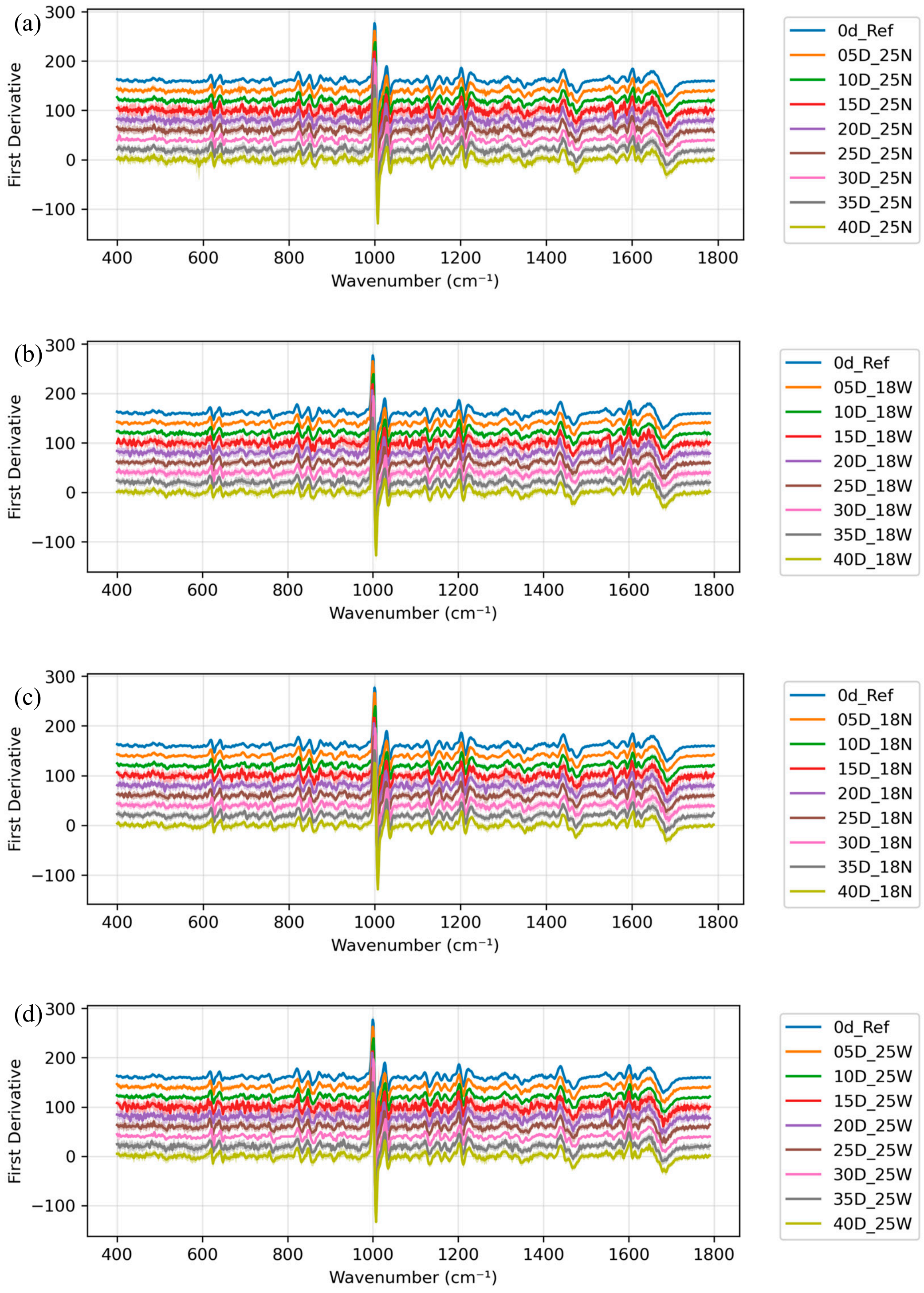

Figure S4 First derivative spectra of egg whites (averaged; with error shades) of all four groups. (**a**–**d**) represent groups L1 to L4, respectively
